# Preliminary behavioral differences between Spanish adults with suicidal ideation and control population

**DOI:** 10.3389/frhs.2025.1630077

**Published:** 2025-11-26

**Authors:** María Carreira Míguez, Ana Isabel Beltrán-Velasco, Eduardo Navarro Jiménez, Vicente Javier Clemente-Suárez

**Affiliations:** 1 Facultad Ciencias Biomédicas y Deporte, Universidad Europea de Andalucía, Malaga, Spain; 2 NBC Group, Psychology Department, School of Life and Nature Sciences, Nebrija University, Madrid, Spain; 3 Facultad de Ciencias de la Salud, Centro de Investigaciones en Ciencias de la Vida, Universidad Simón Bolívar, Barranquilla, Colombia; 4 Grupo de Investigación en Cultura, Educación y Sociedad, Universidad de la Costa, Barranquilla, Colombia; 5 Faculty of Medicine, Health and Sports, Universidad Europea de Madrid, Villaviciosa de Odón, Madrid, Spain

**Keywords:** suicidal ideation, behavioral factors, psychological traits, nutrition, oral health, physical exercise, sleep quality

## Abstract

**Background:**

Suicidal ideation represents a significant public health concern, serving as a crucial predictor of suicide attempts. While biological and psychological risk factors have been thoroughly delineated, the role of daily behaviors such as nutrition, oral health, physical activity, and psychological traits remains to be elucidated.

**Objective:**

This study examined behavioral and psychological differences between Spanish adult's participants with suicidal ideation and a comparison group within a community sample. In Spain, the legal definition of “adult” is primarily determined by the age of majority, which is set at 18 years.

**Methods:**

A total of 1,364 adults from Spain, completed an online survey assessing sociodemographic, anthropometric, nutritional, oral health, physical activity, and psychological variables. Group assignment was based on the Zung Self-Rating Depression Scale, with 20 individuals endorsing suicidal ideation and 1,344 serving as controls. Independent *t*-tests compared groups with a significance level of *p* ≤ 0.05.

**Results:**

Compared with controls, the suicidal ideation group was younger, had lower weight and BMI, reported shorter sleep duration, poorer sleep quality, and more time on social media. They also reported lower water intake and vitality but higher consumption of pastries, protein shakes, and vitamin supplements, along with more frequent migraines and poorer digestion. Oral health findings were counterintuitive, with lower prevalence of gastritis, dry mouth, and dental sensitivity. No significant differences emerged in physical activity. Psychologically, the suicidal ideation group reported greater depression, stress, anxiety, loneliness, and psychological inflexibility, as well as higher neuroticism and openness, and lower extraversion and agreeableness.

**Conclusions:**

Due to the preliminary nature of the study, these findings suggest the presence of distinct behavioral and psychological profiles associated with suicidal ideation. In light of the limited sample size, the single-item classification, and the cross-sectional self-report design, the findings must be regarded as preliminary associations.

## 1 Introduction

Suicide remains a critical global public health issue and a leading cause of premature mortality. The World Health Organization estimates nearly 800,000 suicide deaths occur each year, placing it among the ten leading causes of death worldwide and one of the top three among adolescents and young adults (1). Beyond mortality, suicidal ideation and non-fatal attempts impose substantial psychosocial and economic burdens on individuals, families, and health systems. Within the European Union, suicide continues to represent a major cause of avoidable death, and Spain reports over 4,000 cases annually, prompting the need for effective prevention strategies (2).

Suicidal behavior results from a complex interaction of biological, psychological, and social determinants (3). Depression is among the strongest correlates of suicidal ideation, often exacerbated by social isolation, reduced participation in meaningful activities, and limited social connectedness (4, 5). According to the Interpersonal Theory of Suicide, perceived burdensomeness and thwarted belongingness serve as proximal psychological antecedents of suicidal thoughts, emphasizing the central role of social integration and perceived support (6). In Spain, research exploring suicide has primarily examined epidemiological trends, geographic patterns, and psychosocial correlates (7–10). However, few studies have comprehensively evaluated the relationship between modifiable behavioral factors such as diet, physical activity, technology use, and oral health and suicidal ideation in adult populations. This represents a significant research gap, given that these daily behaviors are potentially alterable through public health and clinical interventions.

Emerging evidence indicates that unhealthy behavioral patterns including sedentary lifestyles, excessive alcohol and tobacco use, poor nutrition, and problematic technology engagement are associated with higher risks of depression and suicidal ideation (11–15). Conversely, protective behaviors, such as regular physical exercise and balanced diets rich in fruits, vegetables, and essential micronutrients, have been shown to enhance psychological resilience and well-being (16–19). These findings suggest that a behavioral approach to suicide prevention, focusing on lifestyle modification, may complement traditional psychiatric and pharmacological strategies.

The COVID-19 pandemic further exposed the sensitivity of mental health to rapid lifestyle disruptions. Stress, as defined by the World Health Organization, involves physical, emotional, or psychological tension and, when chronic, is detrimental to health (20). Evidence from early pandemic phases documented wide-ranging deteriorations in mental health associated with social distancing and quarantine, with disruptions to work, social relations, and daily routines (21, 22). In this context, Killgore and colleagues reported an approximately 43% increase in suicidal ideation among U.S. adults during the first months of confinement, attributing this rise largely to loneliness and heightened psychological burden; importantly, these observations come from survey data in adults and must be interpreted within that population and methodological context (23).

Despite the growing literature linking behavior and mental health, integrated behavioral profiles simultaneously considering dietary habits, physical activity, oral health, and technology use remain underexplored, particularly in Spain. A multidimensional understanding of these profiles may enhance the identification of individuals at risk and guide the design of multi-component interventions that bridge public health promotion with suicide prevention efforts. Therefore, the present study aims to examine behavioral differences between Spanish adults with suicidal ideation and a control population, focusing on nutritional, oral health, physical activity, and psychological domains. It is hypothesized that individuals with suicidal ideation will exhibit distinct behavioral and psychosocial profiles compared with controls, reflecting modifiable risk factors within a multifactorial model of suicide.

## 2 Methods

### 2.1 Participants

The analysis involved a total of 1,364 voluntary adult's participants from Spain. In Spain, the legal definition of “adult” is primarily determined by the age of majority, which is set at 18 years. The Suicidal Ideation Group (SIG) included individuals with a mean age of 31.9 years (±11.5), mean height of 165.9 cm (±10.0), mean weight of 58.3 kg (±14.4), and mean body mass index (BMI) of 20.9 kg/m^2^ (±3.5). The Comparison Group (CG) comprised participants with a mean age of 39.5 years (±14.5), mean height of 169.7 cm (±10.0), mean weight of 71.9 kg (±16.6), and mean BMI of 25.1 kg/m^2^ (±7.9). The allocation of subjects to groups was determined by their responses to the Zung Depression Scale item, “I feel that others would be better off if I were dead.” Participants who endorsed this item at the highest response level (“Most of the time”) were classified into the SIG, whereas the remainder were assigned to the CG. This item has previously demonstrated a significant correlation with suicidal tendency, supporting its use as a proxy indicator in population-based research (24).

The study was approved by the University Ethics Committee (CIPI/18/074) and conducted in accordance with the Declaration of Helsinki (revised in Brazil, 2013). All participants provided written informed consent. The data were collected anonymously and treated with strict confidentiality. To ensure the safety of the participants, members of the SIG were provided with information regarding mental health resources. When deemed appropriate, they were referred to institutional psychological support services.

### 2.2 Design and procedure

Participants completed a battery of validated questionnaires designed to capture multidimensional profiles across nutritional, physical activity, oral health, and psychological domains. Although the number of instruments may appear extensive, this approach was necessary to comprehensively evaluate behavioral differences between groups. To minimize respondent burden, validated short versions were used, when possible (BFI-10, STAI-6, UCLA-3), and the total administration time was approximately 30 min.

1.2.1. The assessment of psychological constructs was conducted using validated instruments adapted to Spanish-speaking populations. In instances where practicability permitted, brief versions supported by psychometric analysis were selected in order to minimize respondent burden while ensuring the coverage of domains pertinent to an integrated behavioural profile.

− Big Five Inventory—Short Version (BFI-10, Spanish adaptation) (25). This 10-item inventory evaluates five personality dimensions (neuroticism, extraversion, openness, agreeableness, conscientiousness) using a 5-point Likert scale (1 = completely disagree to 5 = completely agree). Example item: “I see myself as someone who is reserved”. Reliability was assessed using the reference pattern from the reliability and validity analysis, with *α* = 0.831.
− State–Trait Anxiety Inventory—Short Form (STAI-6, Spanish version) (26). This abbreviated 6-item measure assesses state anxiety with responses ranging from 1 (not at all) to 4 (very much). Example item: “I feel calm” (reverse coded). The reliability analysis was conducted using Cronbach's alpha, with *α* = 0.831.
− Acceptance and Action Questionnaire II (AAQ-II, Spanish version) (27). This 7-item scale measures psychological inflexibility and experiential avoidance, using a 7-point Likert scale (0 = never true to 6 = always true). Higher scores reflect greater inflexibility. Example item: “Emotions cause problems in my life”). The reliability analysis was conducted using Cronbach's alpha, with *α* = 0.831.
− UCLA Loneliness Scale—Short Form (3 items) (28). This short version assesses perceived loneliness through three items rated on a 3-point Likert scale (1 = never to 3 = frequently). Example item: “My interests and ideas are not shared by those around me”). The reliability analysis was conducted using Cronbach's alpha, with *α* = 0.831.
− Perceived Stress Scale (PSS-14, Spanish version) (29). This 14-item instrument evaluates perceived stress over the past month, using a 5-point Likert scale (0 = never to 4 = very often). Example item: “In the last month, how often have you felt difficulties were piling up so high that you could not overcome them?”). The reliability analysis was conducted using Cronbach's alpha, with *α* = 0.831.
− Zung Self-Rating Depression Scale (SDS, Spanish version) (24). This 20-item scale measures depressive symptoms across somatic, cognitive, mood, and psychomotor domains, rated on a 4-point Likert scale (1 = a little of the time to 4 = most of the time). Example item: “I feel downhearted and blue”). The reliability analysis was conducted using Cronbach's alpha.

#### 2.2.2 Nutritional, oral health, and physical activity measures

− Nutritional patterns were assessed through a questionnaire adapted from previous studies in Spanish university populations (30). The instrument included 25 items covering weekly consumption of major food groups (fruit, vegetables, meat, fish, cereals, dairy products, beverages) and additional questions on supplement use, vitality, and hydration. It also incorporated items on migraine frequency (31) and gastrointestinal function, including digestion quality and stool type, the latter classified according to the Bristol Scale (32).
− Oral health habits were evaluated through items concerning toothbrushing frequency, flossing, dental checkups, and related variables, adapted from prior surveys used in Spanish populations (30).
− Physical activity was measured through self-report questions regarding daily steps, minutes of aerobic activity, and resistance exercise during the previous 7 days, following protocols used in earlier behavioral health studies (33). Prior studies have characterized physical activity patterns in affective disorders through the use of ambulatory assessment paradigms (34).

### 2.3 Statistical analysis

The statistical analysis was carried out using the Statistical Package for the Social Sciences (SPSS) version 21.0 (SPSS Inc., Chicago, IL, USA). Descriptive statistics (means and standard deviations) were obtained for all variables. Between-group differences (Suicidal Ideation Group vs. Comparison Group) were examined with independent *t*-tests, using a two-tailed significance level of *p* ≤ 0.05. Given the unequal group sizes, the analyses should be interpreted as exploratory indicators of potential behavioral differences.

## 3 Results

The descriptive statistics (means ± SD) for all variables are presented in Tables 1–5. The Suicidal Ideation Group (SIG) was found to be significantly younger than the Comparison Group (CG) (31.9 ± 11.5 vs. 39.5 ± 14.5 years, *p* = 0.021). Additionally, the SIG exhibited a lower mean weight (58.3 ± 14.4 vs. 71.8 ± 16.6 kg, *p* < 0.001) and body mass index (20.9 ± 3.4 vs. 25.1 ± 7.9 kg/m^2^, *p* = 0.019). In relation to sleep, the SIG reported a decrease in the number of hours of rest per day (6.3 ± 1.1 vs. 7.0 ± 0.9, *p* = 0.001) and a decline in subjective sleep quality (4.0 ± 2.4 vs. 6.8 ± 1.9, *p* < 0.001). Concurrently, the SIG reported an increase in time spent on social media (6.4 ± 12.3 vs. 2.0 ± 5.8 h/day, *p* = 0.001). No significant differences were observed for either height or television viewing time.

**Table 1:** Anthropometric and sociodemographic variables analyzed.

Variable	Comparison group	Suicidal ideation group	*T*	*p*	Cohen's *D*	Confidence interval	
						Lower	Upper
Age	39.5 ± 14.5	31.9 ± 11.5	2.318	0.021	0.58	1.16264	13.95612
Height	169.7 ± 10.0	165.9 ± 10.0	1.690	0.091	0.38	−.61044	8.21665
Weight	71.8 ± 16.6	58.3 ± 14.4	3.632	0.000	0.87	6.23897	20.89220
Body mass index	25.1 ± 7.9	20.9 ± 3.4	2.346	0.019	0.69	.68153	7.63664
Sleep time (h/day)	7.0 ± 0.9	6.3 ± 1.1	3.187	0.001	0.70	.24854	1.04463
Sleep quality (0–10)	6.8 ± 1.9	4.0 ± 2.4	6.529	0.000	1.29	1.95391	3.63240
Time watching TV (h/day)	2.3 ± 8.5	2.8 ± 4.4	−.264	0.792	−0.07	−4.24176	3.23583
Time in social media (h/day)	2.0 ± 5.8	6.4 ± 12.3	−2.272	0.001	−0.46	−6.98312	−1.74803

**Table 2:** Food consumption frequency of control and suicidal ideation groups.

Portions consumed per week	Comparison group	Suicidal ideation group	*T*	*p*	Cohen's *D*	Confidence interval	
						Lower	Upper
Water glasses per day (250 mL)	4.0 ± 1.3	2.5 ± 1.1	4.913	0.000	1.25	0.29896	0.88241
Week vitality (0–10)	7.2 ± 1.7	5.1 ± 2.	5.602	0.000	1.13	0.37679	1.37153
Vitality at the end of week (0–10)	6.0 ± 2.7	2.7 ± 2.6	6.356	0.000	1.25	0.52598	2.31121
Migraine headache occurrence (0–10)	2.8 ± 3.25	5.0 ± 3.7	−3.072	0.002	−0.63	0.73216	−3.68516
Juice weekly consumption (250 mL)	1.8 ± 1.1	1.6 ± 1.0	0.895	0.371	0.19	0.23801	−0.25385
Distilled alcohol weekly consumption (50 mL)	1.3 ± 0.5	1.0 ± 0.0	2.559	0.011	0.85	0.12767	0.07630
Bear weekly consumption (250 mL)	1.8 ± 1.0	1.4 ± 0.5	1.873	0.061	0.51	0.21452	−0.01901
Wine weekly consumption (50 mL)	1.5 ± 0.7	1.1 ± 0.3	2.413	0.016	0.74	0.16669	0.07528
Soft drink weekly consumption (250 mL)	1.7 ± 1.0	2.1 ± 1.5	−1.923	0.055	−0.31	0.21775	−0.84583
Energy drink weekly consumption (250 mL)	1.2 ± 0.5	1.1 ± 0.3	0.433	0.665	0.24	0.12110	−0.18507
Coffee weekly consumption (250 mL)	2.9 ± 1.6	2.4 ± 1.8	1.172	0.241	0.29	0.38144	−0.30117
Tea weekly consumption (250 mL)	1.7 ± 1.1	1.8 ± 1.4	−0.486	0.627	−0.08	0.25307	−0.61945
Milk weekly consumption (250 mL)	2.7 ± 1.5	2.6 ± 1.4	0.365	0.715	0.07	0.32656	−0.52136
Fermented milk products weekly consumption (125 g-mL)	2.6 ± 1.2	1.9 ± 1.3	−1.227	0.220	0.56	0.27635	−0.88115
Pastries weekly consumption (1 portion)	1.8 ± 0.9	2.2 ± 0.6	−2.067	0.039	−0.52	0.21513	−0.86664
Cookies-sweet cereals weekly consumption (30 g-mL)	1.9 ± 1.1	1.7 ± 0.8	1.004	0.315	0.21	0.24582	−0.23535
Cheese weekly consumption (50 g)	2.5 ± 1.1	2.4 ± 0.9	0.357	0.721	0.10	0.26020	−0.41750
Eggs weekly consumption (1 piece)	2.8 ± 1.0	2.5 ± 0.5	1.007	0.314	0.38	0.24967	−0.23826
Meat weekly consumption (150 g)	2.9 ± 1.0	2.3 ± 0.8	2.226	0.026	0.66	0.24597	0.06510
Fish weekly consumption (150 g)	2.5 ± 0.9	2.0 ± 0.7	2.368	0.018	0.62	0.20733	0.08421
Sausages weekly consumption (50 g)	2.2 ± 1.0	2.2 ± 0.6	0.021	0.983	0.00	0.24639	−0.47810
Legumes weekly consumption (200 g)	2.4 ± 0.9	2.4 ± 1.1	−0.084	0.933	0.00	0.21058	−0.43070
Rice weekly consumption (150 g)	2.4 ± 0.9	2.4 ± 0.9	−0.216	0.829	0.00	0.22316	−0.48589
Pasta weekly consumption (150 g)	2.3 ± 0.9	2.2 ± 1.2	0.432	0.666	0.09	0.21490	−0.32874
Fruits weekly consumption (1 portion)	3.4 ± 1.4	3.1 ± 1.2	0.985	0.325	0.23	0.32277	−0.31526
Fresh Vegetables-Salad weekly consumption (200 g)	3.1 ± 1.3	2.6 ± 0.9	1.749	0.081	0.45	0.30014	−0.06392
Cooked weekly consumption (200 g)	3.1 ± 1.3	2.7 ± 1.0	1.452	0.147	0.34	0.29609	−0.15105
Bread weekly consumption (50 g)	2.9 ± 1.3	3.3 ± 1.4	−1.522	0.128	−0.30	0.31205	−1.08705
Whole grain-rice-oat weekly consumption (30 g)	2.3 ± 1.2	2.8 ± 1.4	−1.790	0.074	−0.38	0.29332	−1.10050
Fast food-pizza- hamburger weekly consumption (1 serving)	1.8 ± 0.7	1.4 ± 0.5	1.850	0.064	0.66	0.16575	−0.01847
Protein shakes weekly consumption (300 mL)	1.4 ± 0.9	2.1 ± 1.8	−3.330	0.001	−0.49	0.21920	−1.15988
Vitaminic supplements weekly consumption (1 capsule)	1.4 ± 0.9	2.7 ± 1.9	−5.692	0.000	−0.87	0.22346	−1.71022
Daily teaspoons of sugar	1.4 ± 0.5	1.2 ± 0.4	1.295	0.196	0.44	0.11939	−0.07960
Post-meal digestion (1–5)	1.5 ± 0.6	2.0 ± 0.9	−3.651	0.000	−0.65	0.14222	−0.79828
Bristol scale	3.6 ± 1.1	3.8 ± 2.3	−0.777	0.437	−0.11	0.25399	−0.69556

**Table 3:** Oral health variables analyzed.

Variable	Comparison group	Suicidal ideation group	*T*	*p*	Cohen's *D*	Confidence interval	
						Lower	Upper
Daily toothbrushing (n°)	2.5 ± 0.9	2.5 ± 0.9	0.154	0.878	0.00	0.19728	−0.35663
Smoker	1.3 ± 0.9	1.3 ± 0.9	0.163	0.870	0.00	0.19204	−0.34536
Gastritis or heartburn	2.6 ± 0.6	1.9 ± 0.9	4.512	0.000	0.92	0.14415	0.36767
Dry mouth	2.5 ± 0.7	1.8 ± 0.8	4.459	0.000	0.93	0.15564	0.38874
Dental sensibility	2.3 ± 0.7	1.6 ± 0.8	4.373	0.000	0.93	0.16816	0.40544
Illness days per year	3.7 ± 11.0	5.6 ± 6.8	−0.783	0.434	−0.21	2.45930	−6.74935

**Table 4:** Physical activity variables analyzed.

Variable	Comparison group	Suicidal ideation group	*T*	*p*	Cohen's *D*	Confidence interval	
						Lower	Upper
Daily steps last week	13,486.0 ± 45,088.6	19,653.0 ± 19,034.2	−0.611	0.542	−0.178	10,101.14972	−25,985.36506
Physical activity last 7 days (h)	1.2 ± 0.4	1.1 ± 03	1.358	0.175	0.047	0.09424	−0.05686
Aerobic activity last 7 days (min)	234.5 ± 480.2	359.8 ± 436.2	−1.036	0.300	−0.273	120.81912	−362.28068
Self-loading last 7 days (min)	115.0 ± 216.6	171.3 ± 195.9	−1.032	0.302	−0.273	54.51717	−163.26042

**Table 5:** Psychological variables analyzed.

Variable	Comparison group	Suicidal ideation group	*T*	*p*	Cohen's *D*	Confidence interval	
						Lower	Upper
Zung	46.9 ± 5.1	55.1 ± 5.1	−6.987	0.000	−1.61	−10.44412	−5.86518
PSS4	5.3 ± 3.2	10.1 ± 3.6	−6.655	0.000	−1.41	−6.27624	−3.41859
STAI	12.5 ± 3.8	18.0 ± 5.1	−6.346	0.000	−1.22	−7.16636	−3.78194
UCLA	4.2 ± 1.5	5.8 ± 1.8	−4.676	0.000	−0.97	−2.21963	−.90769
AAQII	20.3 ± 9.7	37.8 ± 12.2	−7.970	0.000	−1.59	−21.80728	−13.19302
Extraversion	5.8 ± 1.7	4.8 ± 2.1	2.756	0.000	0.52	0.30677	1.82144
Agreeableness	6.6 ± 1.5	5.2 ± 2.0	3.558	0.000	0.79	0.53633	1.85421
Conscientiousness	7.3 ± 1.6	6.9 ± 2.4	1.023	0.306	0.20	−0.34921	1.11051
Neuroticism	5.9 ± 2.0	7.4 ± 2.1	−3.471	0.001	0.73	−2.39694	−.66598
Open to experience	7.2 ± 1.7	8.0 ± 2.4	−2.069	0.039	−0.38	−1.58563	−.04214

Zung, depression scale variable; PSS4, perceives stress scale; STAI, Spielberger state-trait anxiety inventory; UCLA, UCLA loneliness scale; AAQII, acceptance and action questionnaire II; B5, big five factors (extraversion, agreeableness, conscientiousness, neuroticism and openness to experience).

Regarding nutritional habits, the CG reported higher water intake (4.0 ± 1.3 vs. 2.5 ± 1.1 glasses/day, *p* < 0.001). They also reported greater perceived vitality during the week (7.2 ± 1.7 vs. 5.1 ± 2.0, *p* < 0.001) and at the end of the week (6.0 ± 2.7 vs. 2.7 ± 2.6, *p* < 0.001). Additionally, CG reported greater consumption of distilled alcohol (1.3 ± 0.5 vs. 1.0 ± 0.0 portions/week, *p* = 0.011) and wine (1.5 ± 0.7 vs. 1.1 ± 0.3 portions/week, *p* = 0.016), as well as meat (2.9 The mean ± standard deviation of portions per week was 1.0 ± 0.8 for the first group and 2.3 ± 0.8 for the second group (*p* = 0.026). The mean ± standard deviation of portions per week was 2.5 ± 0.9 for the first group and 2.0 ± 0.7 for the second group (*p* = 0.018). In contrast, the SIG reported a higher frequency of migraine episodes (5.0 ± 3.7 vs. 2.8 ± 3.3, *p* = 0.002), more frequent consumption of pastries (2.2 ± 0.6 vs. 1.8 ± 0.9 portions/week, *p* = 0.039), greater intake of protein shakes (2.1 ± 1.8 vs. 1.4 ± 0.9 portions/week, *p* = 0.001), and increased consumption of vitamin supplements (2.7 ± 1.9 vs. 1.4 ± 0.9 portions/week, *p* < 0.001). The SIG also reported poorer post-meal digestion (2.0 ± 0.9 vs. 1.5 ± 0.6, *p* < 0.001). A lack of statistically significant disparities was observed among the consumption patterns of juice, beer, soft drinks, energy drinks, coffee, tea, milk, legumes, rice, pasta, fruits, and vegetables across various demographic groups.

Furthermore, a discernible distinction emerged in the psychological profile. The SIG demonstrated higher outcomes in several domains, including depression (55.1 ± 5.1 vs. 46.9 ± 5.1, *p* < 0.001), perceived stress (10.1 ± 3.6 vs. 5.3 ± 3.2, *p* < 0.001), anxiety (18.0 ± 5.1 vs. 12.5 ± 3.8, *p* < 0.001), loneliness (5.8 ± 1.8 vs. 4.2 ± 1.5, *p* < 0.001), and psychological inflexibility (37.8 ± 12.2 vs. 20.3 ± 9.7, *p* < 0.001). In the personality traits, the SIG demonstrated higher scores on neuroticism (7.4 ± 2.1 vs. 5.9 ± 2.0, *p* = 0.001) and openness to experience (8.0 ± 2.4 vs. 7.2 ± 1.7, *p* = 0.039), while reporting lower levels of extraversion (4.8 ± 2.1 vs. 5.8 ± 1.7, *p* < 0.001) and agreeableness (5.2 ± 2.0 vs. 6.6 ± 1.5, *p* < 0.001). No significant differences were observed in conscientiousness (6.9 ± 2.4 vs. 7.3 ± 1.6, *p* = 0.306).

## 4 Discussion

The objective of the present study was to examine behavioral and psychological differences between participants reporting suicidal ideation and a comparison group within a large community sample. In accordance with the objectives of the study, individuals who exhibited suicidal ideation demonstrated a distinct profile that was characterized by younger age, lower body mass index, shorter and poorer sleep, greater use of social media, and differences in nutritional habits, in addition to a less favorable psychological profile. These findings suggest that suicidal ideation in the community is associated with a multidimensional constellation of sociodemographic, behavioral, and psychological characteristics. This observation lends support to the notion that when exploring risk markers for suicidal ideation, it is imperative to consider not only psychological variables but also daily lifestyle factors.

The data obtained identified associations between suicidal ideation and a range of sociodemographic, behavioral, and psychological characteristics in a large community sample. Individuals reporting suicidal ideation were found to be younger, had lower body weight and BMI, reported shorter and poorer sleep, and spent more time on social media. A divergence in dietary patterns was also observed, characterized by a reduced consumption of water, meat, fish, and wine, accompanied by an increased intake of pastries, protein shakes, and vitamin supplements. Furthermore, oral health variables exhibited an unexpected pattern, with suicidal ideation being associated with a lower prevalence of gastritis, dry mouth, and dental sensitivity. Due to the modest size of the suicidal ideation group and the cross-sectional, self-report nature of the study, the findings must be regarded as preliminary.

With regard to sociodemographic and sleep-related findings, younger age and lower BMI in the suicidal ideation group are consistent with previous research linking these characteristics with psychological vulnerability (5, 35). Sleep disturbances were also observed, with participants reporting suicidal ideation experiencing both reduced sleep duration and diminished sleep quality. These findings are consistent with the existing body of evidence indicating that sleep disturbances frequently co-occur with mood disorders and suicidal ideation (36), although the directionality of the relationship cannot be ascertained. The increased time spent on social media in the suicidal ideation group aligns with previous reports indicating that problematic or excessive digital engagement is associated with diminished psychological well-being and elevated distress (37).

Nutritional patterns exhibited several disparities. The findings, which included lower water consumption and vitality scores, along with higher reports of digestive discomfort, are consistent with studies that have established a link between hydration and gastrointestinal well-being with psychological status (38). The elevated consumption of pastries, protein shakes, and vitamin supplements among the suicidal ideation group may be indicative of attempts to cope with fatigue or body image concerns. The comparison group exhibited a higher frequency of alcohol, wine, meat, and fish consumption, which is a noteworthy finding. This phenomenon may appear counterintuitive, given the established correlation between alcohol misuse and an elevated risk of suicidal behavior (39). One potential explanation for this observation is that in our sample, lower alcohol intake in the suicidal ideation group could be indicative of avoidance due to comorbid conditions or medication, rather than a protective pattern.

The oral health variables demonstrated a reduced prevalence of gastritis, dry mouth, and dental sensitivity in the suicidal ideation group compared to the comparison group. This finding stands in contrast to the findings of much of the extant literature, which reports a higher prevalence of oral and gastrointestinal problems among individuals with depressive symptoms or suicidal behaviors (40). Several factors may contribute to these results. Differences in demographic and socioeconomic characteristics could have influenced the oral health outcomes. It is also possible that individuals experiencing suicidal ideation may place greater emphasis on reporting psychological symptoms while downplaying somatic or oral complaints, leading to potential reporting bias.

With respect to physical activity, no significant differences were detected between the groups. These data are not in accordance with diverse findings suggesting that regular physical activity is associated with reduced levels of depression and suicidal ideation (41, 42). The absence of group differences in the present study may be attributable to the limited statistical power of the suicidal ideation group, variability in self-reported activity, or the relatively broad nature of the questions included.

The psychological profile of participants with suicidal ideation was consistent with initial hypothesis. The subjects exhibited elevated levels of depression, stress, anxiety, loneliness, and psychological inflexibility, along with heightened neuroticism and openness, and diminished extraversion and agreeableness. These findings align with established models of personality and psychopathology, which have demonstrated a persistent association between neuroticism and emotional instability, as well as suicide risk. Conversely, low extraversion and agreeableness have been linked to diminished social connectedness and greater interpersonal difficulties (43, 44). The elevated openness exhibited by the suicidal ideation group may appear counterintuitive at first glance. However, analogous patterns have been documented in specific community samples (19). This result could be indicative of heightened sensitivity to internal experiences, creative tendencies, or contextual factors not adequately addressed in this study. Nevertheless, the study underscores the heterogeneity of psychological profiles among individuals reporting suicidal ideation.

## 5 Limitations of the study and future research directions

It is imperative to acknowledge the limitations of this study when interpreting the findings. Initially, the Suicidal Ideation Group (SIG) was modest in size, with a total of 20 participants. This reduced the statistical power of the study and constrained the extent to which the findings could be generalized. However, the overall sample size remained considerable. Secondly, the cross-sectional and self-report design of the study imposes limitations on the inferences that can be drawn regarding causality, and it may introduce biases related to recall or reporting. Thirdly, the group assignment was predicated on a solitary item from the Zung Self-Rating Depression Scale, which, while having been previously utilized in large-scale surveys, does not encapsulate the multidimensional nature of suicidal ideation. It is therefore recommended that future studies incorporate validated multi-item scales, such as the Beck Scale for Suicidal Ideation (BSSI) or the Columbia-Suicide Severity Rating Scale (C-SSRS). Fourthly, relevant confounders, including socioeconomic status, medication use, and psychiatric comorbidities, were not assessed and should be systematically considered in future research. Fifthly, the statistical analyses were exploring in nature, employing multiple univariate comparisons without the application of a correction for multiple testing.

Future research should address these limitations by recruiting larger and more balanced samples, using validated measures of suicidal ideation, incorporating comprehensive covariates, and applying multimethod and longitudinal designs. The integration of objective assessments (e.g., actigraphy, clinical interviews) and the testing of targeted interventions on modifiable behavioral factors may further advance understanding and prevention. Multidisciplinary approaches encompassing psychology, psychiatry, nutrition, and public health remain imperative.

## 6 Practical applications

The findings of this exploratory study may have practical implications for both clinical and public health settings. Healthcare providers may consider the identified behavioural and psychological profiles as potential markers to improve screening and to identify individuals who may be at elevated risk of suicidal ideation. These profiles have the potential to inform the development of early, customized interventions that address sleep quality, nutritional habits, and psychological flexibility, thereby supporting more personalized care. At the community level, public health strategies could incorporate these insights into awareness campaigns and preventive programs, emphasizing the importance of healthy lifestyle behaviours and psychological well-being as part of suicide prevention efforts. Although further validation is required, the present results offer a basis for designing multidimensional approaches that integrate behavioural and psychological domains into risk assessment and intervention planning.

## 7 Conclusion

The present study identified distinct behavioral and psychological profiles associated with suicidal ideation in a large community sample. Individuals reporting suicidal ideation were found to be younger, had lower body mass index, shorter and poorer sleep, and greater social media use. These individuals also exhibited differences in dietary habits and a less favorable psychological profile. No discrepancies were detected in physical activity, yet certain outcomes, including diminished oral health complaints, proved unanticipated and necessitate additional scrutiny. While the findings must be interpreted with caution due to the study's limited sample size, reliance on a unidimensional classification system, and cross-sectional design, they offer preliminary evidence that suicidal ideation may co-occur with a multidimensional set of lifestyle and psychological factors. The insights obtained demonstrate the necessity of incorporating behavioral, nutritional, and psychological dimensions into future research and preventive strategies.

## Data Availability

The raw data supporting the conclusions of this article will be made available by the authors, without undue reservation.
